# Characterization of Chicken Tumor Necrosis Factor-α, a Long Missed Cytokine in Birds

**DOI:** 10.3389/fimmu.2018.00605

**Published:** 2018-04-17

**Authors:** Franziska Rohde, Benjamin Schusser, Tomáš Hron, Helena Farkašová, Jiří Plachý, Sonja Härtle, Jiří Hejnar, Daniel Elleder, Bernd Kaspers

**Affiliations:** ^1^Department of Veterinary Science, Ludwig-Maximilians-Universität, Munich, Germany; ^2^Reproductive Biotechnology, Department of Animal Sciences, Technical University Munich, Munich, Germany; ^3^Laboratory of Viral and Cellular Genetics, Institute of Molecular Genetics of the Czech Academy of Sciences, Prague, Czechia

**Keywords:** tumor necrosis factor-α, chicken, avian, tumor necrosis factor-α receptors, missing gene, biological activity

## Abstract

Tumor necrosis factor-α (TNF-α) is a pleiotropic cytokine playing critical roles in host defense and acute and chronic inflammation. It has been described in fish, amphibians, and mammals but was considered to be absent in the avian genomes. Here, we report on the identification and functional characterization of the avian ortholog. The chicken TNF-α (chTNF-α) is encoded by a highly GC-rich gene, whose product shares with its mammalian counterpart 45% homology in the extracellular part displaying the characteristic TNF homology domain. Orthologs of chTNF-α were identified in the genomes of 12 additional avian species including *Palaeognathae* and *Neognathae*, and the synteny of the closely adjacent loci with mammalian TNF-α orthologs was demonstrated in the crow (*Corvus cornix*) genome. In addition to chTNF-α, we obtained full sequences for homologs of TNF-α receptors 1 and 2 (TNFR1, TNFR2). chTNF-α mRNA is strongly induced by lipopolysaccharide (LPS) stimulation of monocyte derived, splenic and bone marrow macrophages, and significantly upregulated in splenic tissue in response to i.v. LPS treatment. Activation of T-lymphocytes by TCR crosslinking induces chTNF-α expression in CD4^+^ but not in CD8^+^ cells. To gain insights into its biological activity, we generated recombinant chTNF-α in eukaryotic and prokaryotic expression systems. Both, the full-length cytokine and the extracellular domain rapidly induced an NFκB-luciferase reporter in stably transfected CEC-32 reporter cells. Collectively, these data provide strong evidence for the existence of a fully functional TNF-α/TNF-α receptor system in birds thus filling a gap in our understanding of the evolution of cytokine systems.

## Introduction

Work in mammals over the last 40 years identified members of the tumor necrosis factor (TNF)/TNF receptor (TNFR) superfamilies as critical regulators of diverse biological functions, such as inflammation, immune defense, tissue development, and lymphocyte homeostasis (1–3). The founding member of this family was initially described as a serum factor induced by lipopolysaccharide (LPS) which caused hemorrhagic necrosis of induced fibrosarcomas, hence named TNF (4). Today, more than 40 members of the TNF/TNFR superfamilies have been described (5). TNF family members are type II transmembrane proteins which form trimers either as membrane bound proteins or as soluble factors released from the cell membrane after proteolytic cleavage (6, 7). TNF-ligands bind to one or several receptors of the TNFR family, which are type I transmembrane proteins characterized by their cysteine-rich domains (5, 8, 9).

The identification, cloning, and functional characterization of avian cytokines and their receptors have been hampered by their low sequence homology with their mammalian orthologs, the lack of cross-reactivity of diagnostic tools and of suitable bioassays. Progress was made when the first chicken genome sequence was released (10, 11). Since then several cytokine and cytokine receptor families have been identified mainly in chickens (12) and to some extend in other avian species (13). The first comprehensive analysis of the chicken TNF/TNFR superfamilies identified numerous members but indicated a reduced complexity in comparison with the mammalian system (12). Several functionally important members of this superfamily seemed to be absent from the avian genome as previously observed for other avian cytokine and chemokine families. Most notably, neither TNF-α nor lymphotoxin-α (LTα) or LTβ in birds were found despite their essential roles in immune defense and lymphoid organ development in mammals (11). Despite significant efforts using conventional database analysis and EST database screening (12) as well as expression cloning approaches a chicken TNF-α (chTNF-α) ortholog could not be identified. Furthermore, while the mammalian TNF-α gene is located within the MHC class III region in a cluster with LTα and LTβ, the syntenic region is absent from the chicken MHC locus (14). TNF-α-like biological activities in conditioned media have been reported repeatedly (15–18) but none of these activities could be further characterized and clearly attributed to a TNF-α-like protein. These observations led some authors to conclude that TNF-α is indeed lacking in birds and arguments were put forward that other TNF family members such as TNF-like ligand 1A might at least partially substitute for its absence (11, 19) as discussed for other missing genes (20).

However, TNF-α homologs have been described in the genomes of several teleosts including Japanese flounder, rainbow trout, and common carp (21–23). Functional studies demonstrated homologs biological activities to mammalian TNF-α (23, 24) and characterized fish TNF-α as a potent inducer of inflammatory cytokines and antimicrobial peptides (25). These findings would predict that TNF-α is a phylogenically “old” cytokine which might have been lost during the evolution of avian species (20) or translocated in the genome due to extensive reassortment as observed in the chicken MHC locus and thus escaped identification as a consequence of incomplete shotgun sequences (12). Evidence in favor of the latter hypothesis comes from the identification of orthologs of the two mammalian TNF-α receptors in the chicken genome. In mice and man, TNFR1 is expressed on most cells and binds TNF-α in its membrane bound and soluble forms. Expression of TNFR2 is largely restricted to immune cells and endothelial cells and primarily binds to transmembrane TNF-α (5, 26). Likewise, the chicken TNFR1 ortholog is expressed in a wide variety of tissues (27, 28). By contrast, the chicken TNFR2 ortholog, which was identified through a suppressive subtractive hybridization approach in LPS stimulated spleens, is primarily expressed in lymphoid tissues (29). This receptor shows 31 and 28% homology with its human and murine counterparts, respectively. Using reciprocal BLAST analysis and examination of conserved syntenic regions Kaiser et al. confirmed the presence of a TNFR2 ortholog in the chicken genome (12).

Recently, other “missing” genes were identified in the chicken genome including cytokines, such as erythropoietin (30) and leptin (31, 32) by making use of the increasing number of avian genome sequences and advanced data mining technologies. It turned out that these newly identified genes were highly GC-rich, which for technical reasons caused their absence from genome assemblies and other genetic databases (30). Such genes with high GC content and long GC-rich stretches are very hard to amplify by PCR and are also extremely underrepresented in next generation sequencing data. Thus, based on our previous success with EPO and leptin we set out to search for avian TNF-α. Independently, another group recently reported partial sequence homologous to chTNF-α (33). Here, we report on the assembly of a full-length gene resembling chTNF-α and provide functional evidence that the identified gene is indeed the avian ortholog of mammalian TNF-α. Thus, our study closes a long existing gap in avian cytokine research and in the evolutionary tree of the TNF family.

## Results

### Identification of chTNF-α

**Figure 1:**
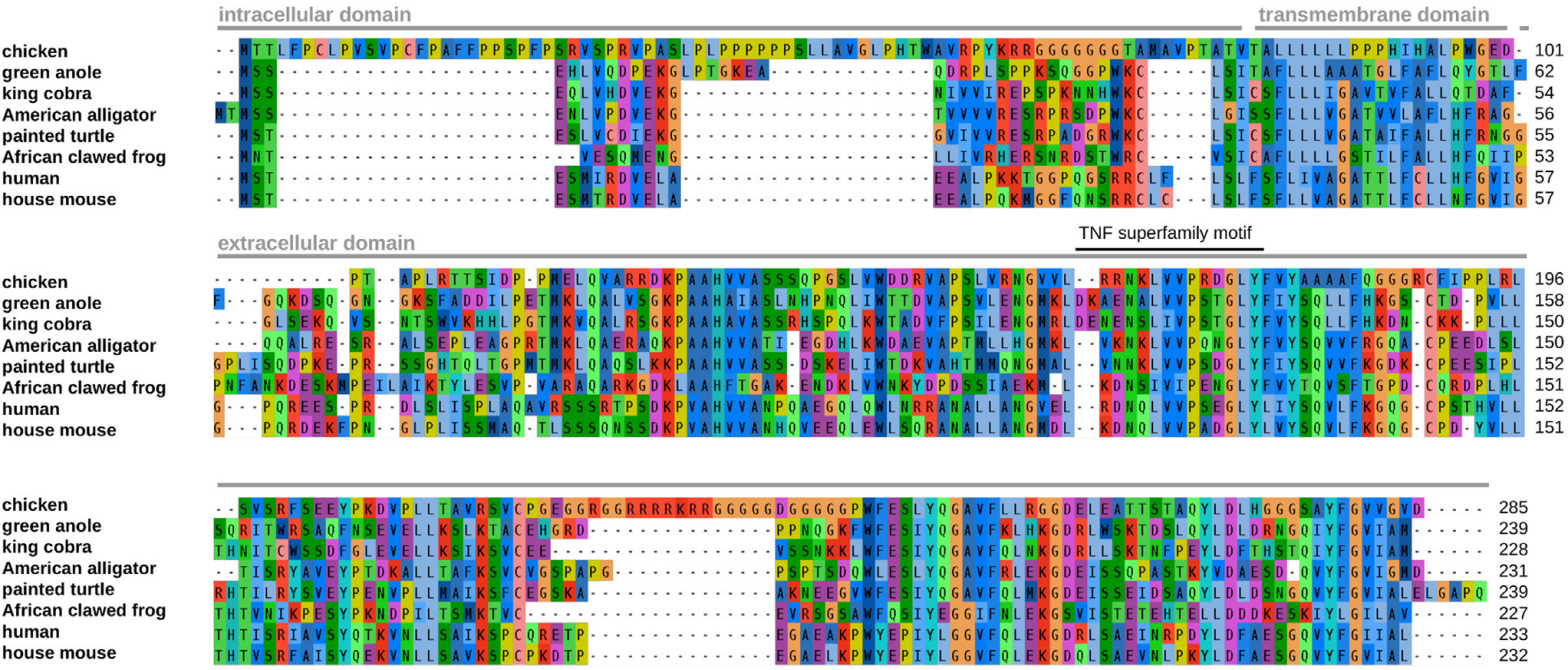
Alignment of tumor necrosis factor (TNF)-α amino acid sequences from chicken and other vertebrates. The positions of predicted protein domains and of the TNF superfamily motif are indicated above the alignment. The transmembrane domain of the chicken protein was predicted using the THMM 2.0 server (http://www.cbs.dtu.dk/services/TMHMM/). The GenBank accession numbers of the TNF-α proteins are the following: green anole (*Anolis carolinensis*; XP_008103955), king cobra (*Ophiophagus hannah*; ETE57607), American alligator (*Alligator mississippiensis*; XP_006258274), painted turtle (*Chrysemys picta bellii*; XP_008175031), African clawed frog (*Xenopus tropicalis*; NP_001107143), human (*Homo sapiens*; AAA61198), and house mouse (*Mus musculus*; AAB65593).

We were able to identify chTNF-α using BLAST searches and manual assembly from Illumina sequence datasets available at the Sequence Read Archive (SRA) of the National Center for Biotechnology Information (NCBI) (see Materials and Methods). According to our previous experience with the discovery of novel chicken GC-rich genes, the key requirement for successful assembly is the use of very large Illumina sequence datasets. This compensates for the extreme underrepresentation bias of these GC-rich regions in the data. The entire coding sequence obtained *in silico* was verified by RT-PCR amplification from chicken RNA. Since GC-rich sequences are hard to amplify, the PCR was carried out in five overlapping shorter fragments that cover the entire chTNF-α coding sequence. The resulting chTNF-α sequence was submitted to GenBank under accession number MF000729. It is predicted to encode a protein of 285 amino acids (Figure 1). Comparison with available sequences from different reptiles and amphibian reveals an extended intracellular domain and a relatively well-conserved extracellular domain. The extracellular domain is well alignable with human (45% similarity) and other vertebrate domains and contains the TNF superfamily motif.

### Phylogenetic Analysis of TNF-α From Chicken and Other Birds

We next used the chTNF-α sequence as a probe in BLAST searches of NCBI SRA datasets from additional avian species. We were able to assemble full or partial TNF-α sequences from a wide variety of avian species (Figure S1 in Supplementary Material) including members of the suborders of *Palaeognathae* and *Neognathae* (with several *Galloanserae* and *Neoaves*). As in the previous examples of GC-rich avian “missing” genes, all avian TNF-α sequences have a high GC content, in contrast to their various non-avian vertebrate orthologs (Figure S2 in Supplementary Material).

**Figure 2:**
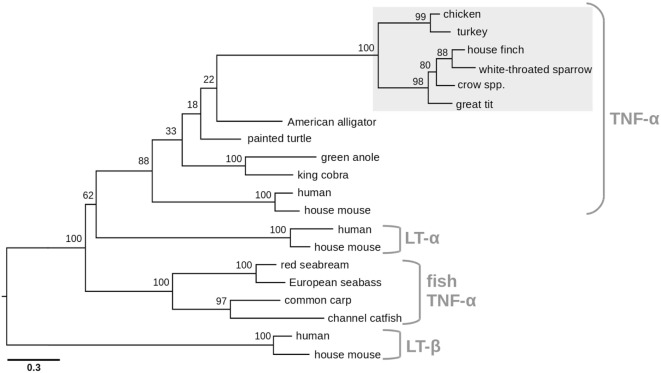
Phylogenetic relationship of avian tumor necrosis factor (TNF)-α proteins with other members of the TNF superfamily. The maximum likelihood tree was generated from avian TNF-α proteins for which full-length sequences were obtained in this study, together with selected vertebrate TNF-α proteins and representatives of lymphotoxin-α (LT-α) and lymphotoxin-β (LT-β) families. TNF-α proteins include the representatives presented in Figure 1, along with avian sequences from this study (turkey, *Meleagris gallopavo*; house finch, *Haemorhous mexicanus*; white-throated sparrow, *Zonotrichia albicollis*; crow spp., *Corvus* spp.; great tit, *Parus major*) and fish sequences: red seabream (*Pagrus major*; AAP76392), European seabass (*Dicentrarchus labrax*; AAZ20770), common carp (*Cyprinus carpio*; CAC84641), and channel catfish (*Ictalurus punctatus*; NP_001187101). LT-α sequences include human (NP_000586) and house mouse (NP_034865), LT-β include human (NP_002332) and house mouse (NP_032544). Bootstrap support values are shown for each node. The scale bar indicates the number of amino acid substitutions per site.

Phylogenetic analysis clearly shows the expected relationship of all newly identified avian TNF-α sequences, reflecting the evolutionary relationship of the species (Figure 2). The avian sequences form a distinct cluster away from fish, reptile, mammalian TNF-α, and the mammalian LT-α and LT-β. We were not able to identify orthologs of LTs in the available avian genomes and other sequence data.

**Figure 3:**
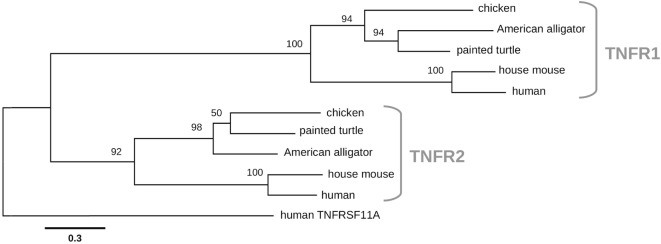
Chicken tumor necrosis factor-α receptors 1 and 2 (TNFR1 and TNFR2) phylogeny. Maximum likelihood tree was constructed using sequences of TNFR proteins from chicken and other vertebrates. The TNFR1 sequences include chicken (NP_001025950), American alligator (XP_019331863), painted turtle (XP_008170508), house mouse (NP_035739), and human (NP_001056). The TNFR2 sequences include chicken (NP_989770), painted turtle (XP_005292860), American alligator (XP_006264541), house mouse (NP_035740), and human (NP_001056). The human TNFRS11A (NP_003830) member of the TNFR superfamily was used as an outgroup. The scale bar indicates the number of substitutions per site.

To confirm the identity of both chicken TNF receptors, we performed phylogenetic analysis of these two genes with the receptors from other vertebrates. Both chicken candidate TNFR1 and TNFR2 (Figure 3) receptors cluster with high bootstrap support with the corresponding TNF receptors from reptiles and mammals (29, 34).

### TNF-α Has Been Translocated in the Avian Genome With Large Genomic Region From the MHC Locus

**Figure 4:**
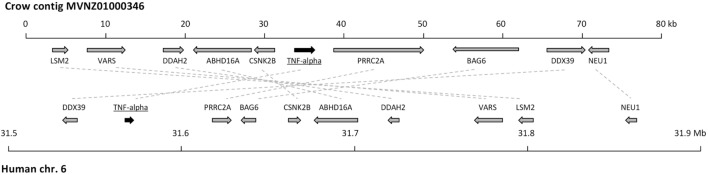
Comparison of syntenic gene order in human and crow tumor necrosis factor (TNF)-α loci. A schematic representation of the crow (upper) and human (lower) chromosomal regions containing TNF-α is shown. The genes are depicted as arrows, approximately to scale, pointing in the direction of transcription. The positions in the human genomic locus correspond to the numbering in the genome release hg38. The candidate orthologous genes from human and crow are connected with dotted lines. The identity of the crow genes was determined by BLAST analysis, in which the corresponding human gene scored as best BLAST hit to the crow sequence.

We attempted to analyze the location of avian TNF-α in broader genomic context. We were not able to assemble the genomic region surrounding the chicken gene. However, in another avian genome, a newly released assembly of the crow (35), we succeeded in the identification of an 80-kb contig (accession number MVNZ01000346) that contained a clear match to our identified avian TNF-α sequence. Importantly, this contig also contained nine additional genes, the orthologs of which lie close to human TNF-α in the MHC locus (Figure 4). Most of the nine genes do not have annotations in any avian genome, to date. This conserved synteny suggests that possibly a chromosomal fragment containing several genes of the MHC complex, including TNF-α, was displaced to a new location in the avian ancestor. The verification of this hypothesis and determination of the chromosomal position would require much more complete genomic assemblies from multiple avian species. The crow genomic sequence also allowed us to determine that the TNF-α gene of this species has the same four-exon structure as in other vertebrates (data not shown).

### chTNF-α Is Induced in Cultured Macrophages, CD4^+^ T-Lymphocytes, and Spleen Tissue

**Figure 5:**
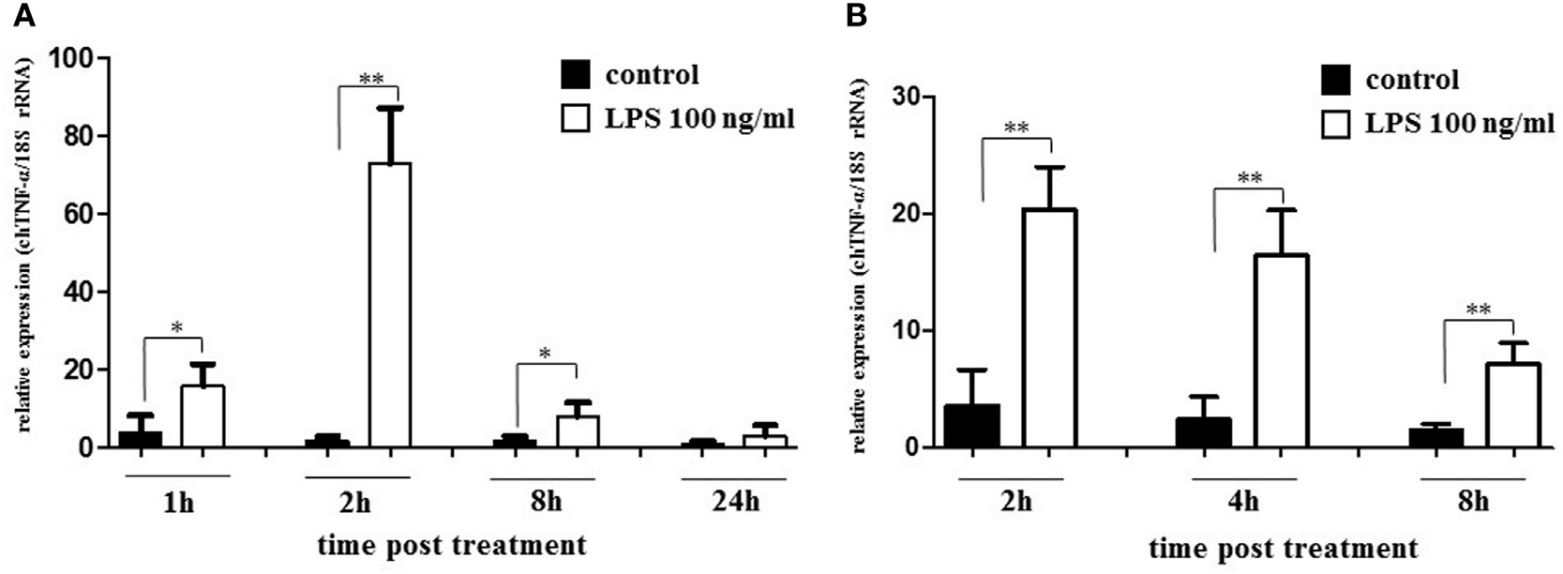
Relative expression of chicken TNF-α (chTNF-α) mRNA in lipopolysaccharide (LPS) stimulated monocyte derived and splenic macrophages. Macrophages isolated either from blood monocytes **(A)** or spleen tissue **(B)** by plastic adherence were stimulated with LPS (100 ng/ml) for the indicated periods. Control macrophages remained unstimulated. Relative expression levels of chTNF-α were analyzed by qRT-PCR. Data represent three independent experiments, significant differences between controls and stimulated cells are indicated (*p ≤ 0.05; **p < 0.01; Student’s *t*-test).

To gain insight into the expression of chTNF-α, we first performed *in vitro* experiments with LPS activated monocyte-derived macrophages. Macrophages are well known from the mammalian system as primary producers of TNF-α. Chicken monocyte-derived macrophages were cultured in the presence of 10 µg/ml or 100 ng/ml of LPS for up to 24 h, lysed and tested for cytokine expression by quantitative RT-PCR. A strong induction of chTNF-α message was observed within 4 h after LPS stimulation with 100 ng/ml being more potent than 10 µg/ml (data not shown). Therefore, subsequent experiments were performed with a final concentration of 100 ng/ml LPS. Monocyte-derived macrophages (>92% KUL01^+^) (Figure 5A) showed significant transcription of chTNF-α (three independent experiments) within 1 h of stimulation and maximal response after 2 h. mRNA levels rapidly declined after 8 h and were at background levels at 24 h. Splenic macrophages (containing > 90% KUL01 positive cells) (Figure 5B) showed similar kinetics with strong induction of chTNF-α message 2, and 4 h after LPS treatment and a reduction at 8 h (three independent experiments). Finally, macrophages differentiated from bone marrow (BM) precursors by colony-stimulating factor 1 (CSF-1) treatment (36) responded equally to LPS but with a slightly delayed onset (Table 1).

**Table 1:** Relative expression of chTNF-α and chIFN-γ mRNA.

Cell type	Stimulated with	rel. Expression chTNF-α	rel. Expression IFN-γ
Lymphocytes	Control	2.4	0.5
	TCR-2	37.5	179.8

CD4^+^ cells	Control	1.1	1.1
	TCR-2	45.7	176.7

CD8^+^ cells	Control	0.6	1.0
	TCR-2	1.1	0.1

Macrophages (BM)	2 h control	2.4	
	2 h LPS	3.0	
	4 h control	0.8	
	4 h LPS	44.4	
	8 h control	1.0	
	8 h LPS	38.2	

*Lymphocytes were obtained from chicken spleen and either stimulated as a mixed population or after FACS sorting to obtain >99% pure CD4^+^ or CD8^+^ T-cells preparations. Cells were stimulated by crosslinking of the α/β-TCR with the monoclonal antibody TCR-2 for 14 h. chIFN-γ mRNA expression was used as a positive control. CSF-1 expanded BM-derived macrophages were stimulated with 100 ng/ml LPS for 2, 4, and 8 h. Relative expression was calculated using 18S rRNA as reference*.

*chTNF-α, chicken TNF-α; LPS, lipopolysaccharide; CSF-1, colony-stimulating factor 1; BM, bone marrow*.

In addition to macrophages, we tested lymphocytes for their capability to produce chTNF-α. Lymphocytes isolated from spleen tissue were subjected to crosslinking of the α/β T-cell receptor (TCR α/β) for 14 h which proved to induce high levels of chIFN-γ mRNA in previous experiments (37) and let to strong induction of chTNF-α message as shown here (Table 1). To identify the chTNF-α producing T-cell subpopulation we sorted in an independent experiment CD4^+^ and CD8^+^ lymphocytes from spleen by FACS sorting to >99% purity and subjected these cells to TCR α/β crosslinking. Strikingly, no response was observed in the CD8^+^ population while T-helper cells transcribed both chTNF-α and chIFN-γ mRNA.

**Figure 6:**
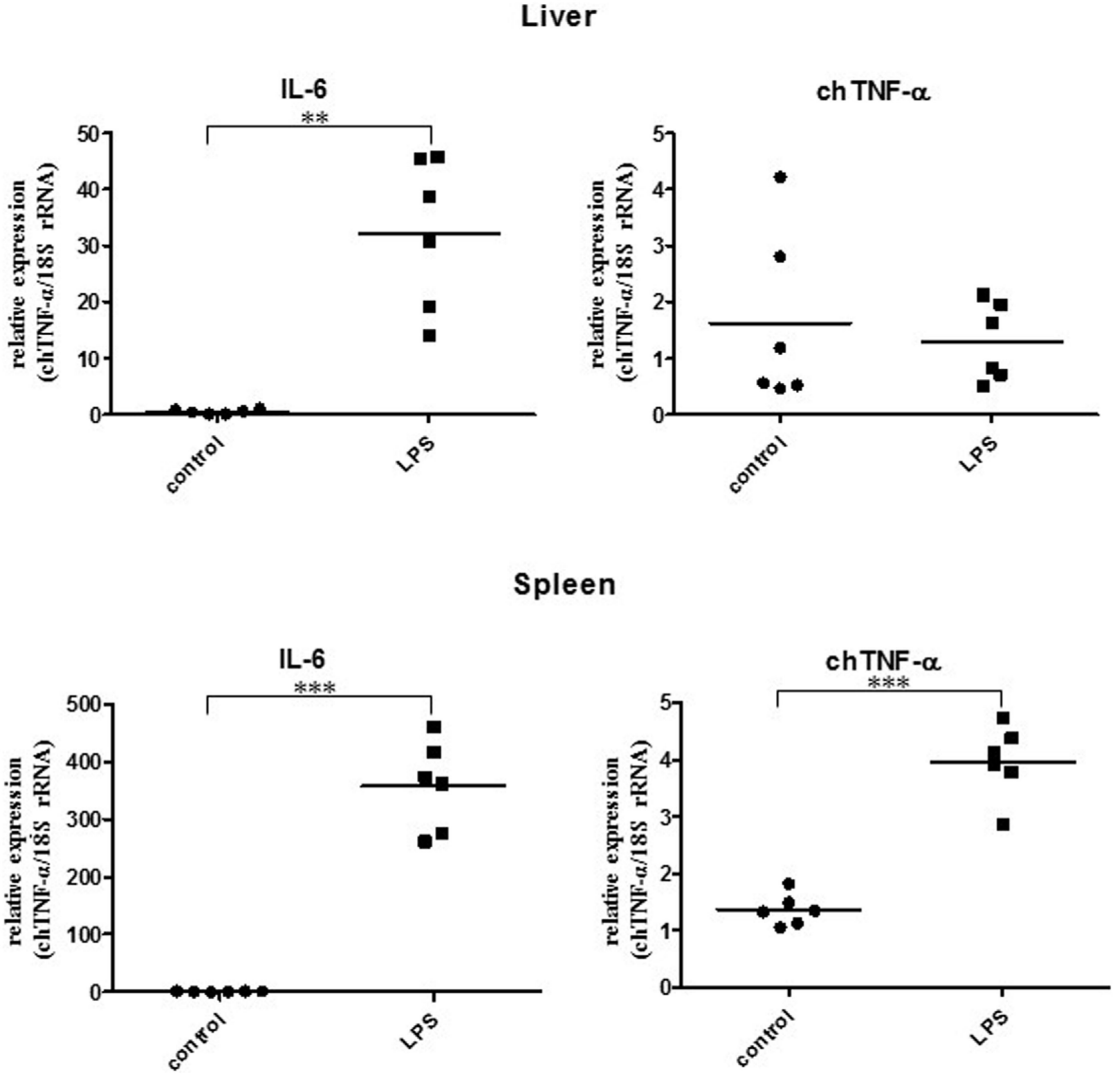
Relative expression of chicken TNF-α (chTNF-α) and chicken interleukin-6 mRNA in liver and spleen of lipopolysaccharide (LPS) treated and control chicken. Animals were treated with 10 µg LPS/kg BW in PBS for 3 h (*n* = 6). Control birds received PBS only (*n* = 6). Cytokine expression was quantified by qRT-PCR. Expression of chTNF-α in response to LPS stimulation in the liver was not significantly different compared to the controls. **p < 0.01; ***p < 0.001 (Student’s *t*-test).

We next injected LPS i.v. into white leghorn hens at a dose of 10 µg/kg BW and collected liver and spleen tissues 3 h after treatment. To compare the chTNF-α response with that of other inflammatory cytokines described in chickens, we quantified chicken interleukin-6 (chIL-6) mRNA in parallel (Figure 6). In liver samples, IL-6 was upregulated 30-fold within 3 h confirming the induction of an acute-phase response in the birds. By contrast, no significant difference was observed for chTNF-α message in liver tissue between non-stimulated control birds and birds treated with LPS. Spleen tissue showed an even stronger induction of chIL-6 mRNA with a 350-fold increase at the same time point. We observed chTNF-α induction in the same samples but to a much lower level with only fourfold upregulation relative to the controls.

Collectively the expression data confirm a rapid induction of chTNF-α in response to toll-like receptor (TLR)-4 ligation both *in vitro* and *in vivo* identifying this cytokine as a typical acute-phase protein.

### Recombinant chTNF-α Is Biologically Active

**Figure 7:**
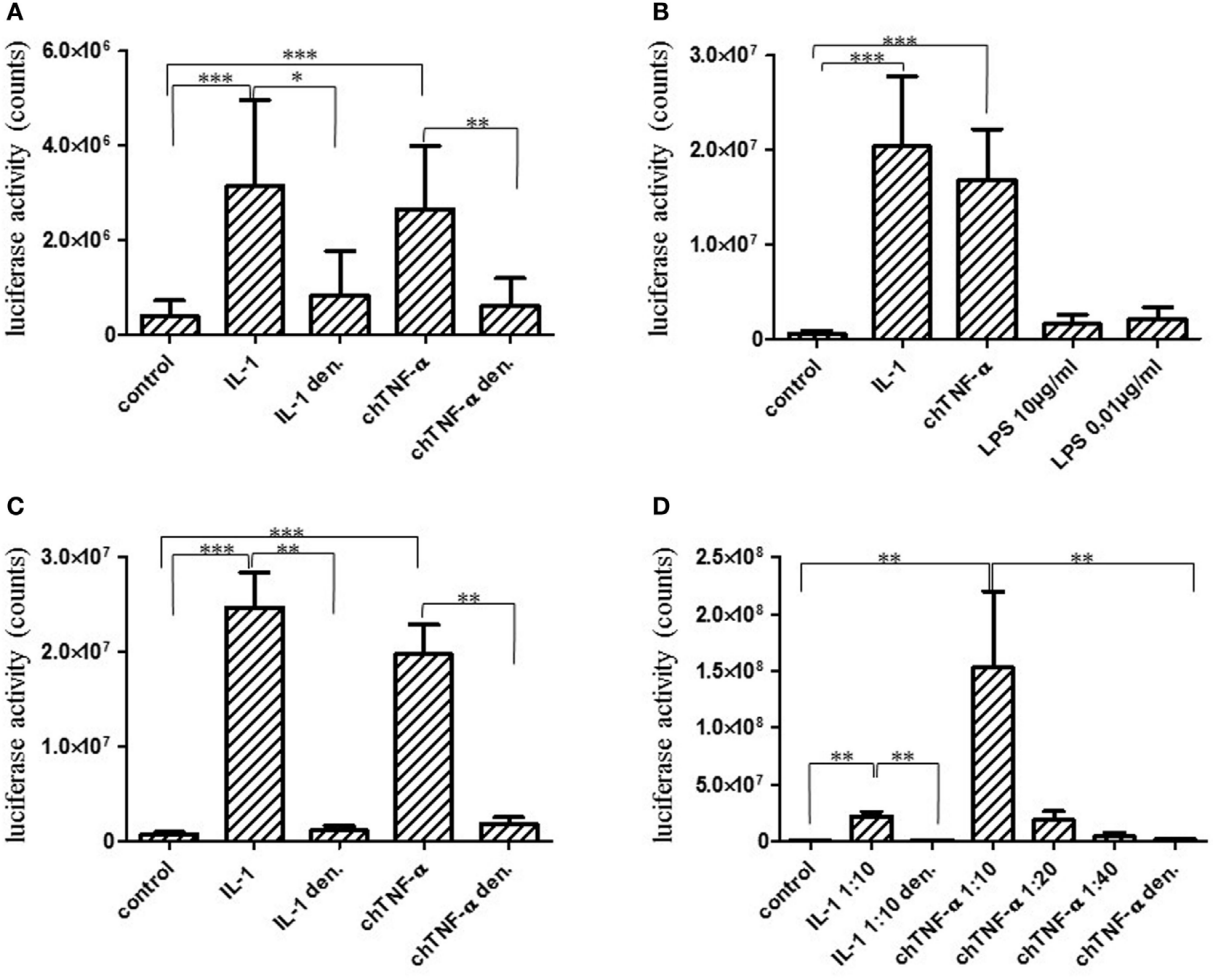
Biological activity of chicken TNF-α (chTNF-α). The full-length TNF-α gene was expressed in HEK293 cells and concentrated cell culture supernatants were added to CEC–NFκB–LUC reporter cells at a 1:25 dilution for 6 h. Biological activity was quantified using a luciferase reporter assay. Luciferase activity is expressed as counts. COS cell expressed chicken interleukin (chIL)-1 was used as a positive control (38). Cytokine preparations were denatured (den.) by heating to 80°C for 5 min. Results represent three independent experiments **(A)**. Full-length chTNF-α **(B,C)**, and the extracellular domain of chTNF-α **(D)** were expressed in *E. coli* and purified by affinity chromatography. Biological activity was quantified as described in **(A)**. *E. coli* lipopolysaccharide (LPS) was added to the reporter cells in parallel to test their sensitivity to potentially contaminating LPS **(B)**. *E. coli* expressed chTNF-α or control chIL-1β were heated to 80°C for 5 min to inactivate the protein **(C)**. Reporter cells were activated with different dilutions of the extracellular domain of chTNF-α and the biological activity quantified as above **(A)**. Results shown in **(A–D)** each represent three independent experiments. *p ≤ 0.05; **p < 0.01; ***p < 0.001 (Mann–Whitney *U*-test, Student’s *t*-test).

Finally, we intended to investigate if the newly identified cytokine is biologically active. Therefore, chTNF-α was synthesized after codon optimization to avoid GC-rich regions for expression in HEK 293 cells and cloned into the expression vector pcDNA3.1 for transient expression. Cell culture supernatants from chTNF-α transfected cells and non-transfected cells were harvested after 12–16 h and added to the quail CEC-NFκB-luciferase reporter cells at a 1:25 dilution. These cells were initially established as reporters for chIL-1 bioactivity. Therefore, supernatants from HEK293 cells transfected with a chIL-1 expression construct served as positive controls. As shown in Figure 7A, rec. chTNF-α activated the reporter with a similar potency as rec. chIL-1. This activity was largely eliminated when samples were heated to 80°C for 5 min thus excluding the possibility that the observed activity is due to contaminating LPS. The presence of recombinant chTNF-α in the culture supernatant was determined by mass spectrometry measurements (see Materials and Methods). Three unique peptides from the chTNF-α protein were detected, with sequence coverage 10.2, MaxQuant Score 83.5 and Q-value (false-positive probability) equal to zero (Figure S3A in Supplementary Material).

In addition, we expressed chTNF-α in *E. coli* as a full-length version and as a version encompassing only the extracellular domain and purified them by Ni-chelate chromatography. Proper expression of the extracellular portion was confirmed by Western blotting identifying a His-tagged protein band at the correct molecular size (Figure S3B in Supplementary Material). As shown in Figure 7B, the full-length chTNF-α strongly induced the luciferase reporter. This effect was not due to LPS contamination since LPS added at high (10 µg/ml) and low (10 ng/ml) concentrations to the cells did not lead to reporter induction (Figure 7B). In addition, LPS concentrations in our cytokine preparations were found to be 0.7 ng/ml at maximum as quantified by Limulus amebocyte lysate (LAL) assay and thus well below those concentrations tested on the reporter cells. Finally, the activity was completely abrogated by heating to 80°C for 5 min (Figure 7C). Likewise, the extracellular version of chTNF-α highly significantly activated the reporter cells. This biological activity was completely abrogated if chTNF-α was heat treated prior to addition to the cell culture (Figure 7D).

## Discussion

TNF-α is a pleiotropic cytokine essential to control intracellular bacteria, such as *Listeria monocytogenes, Mycobacterium tuberculosis*, and *Salmonella typhimurium* as shown in TNF receptor 1 deficient mice (2). It is also known to be an important endogenous mediator of acute (39) and chronic inflammatory (40) responses and is successfully targeted to treat rheumatoid arthritis in humans (41, 42). While orthologs of TNF-α were identified in many mammalian species, amphibian, and teleost fish (25, 43, 44), it seemed to be absent in avian species. Using very large Illumina sequence datasets, we were able to assemble a gene which showed significant homology to TNF-α and to further characterize it as a true ortholog by its genomic structure and its biological properties.

Chicken TNF-α, like other “missing genes” in avian species, escaped from earlier identification due to its GC richness and the absence of readily identifiable syntenic regions. A most recently published study newly described 137 genes classified as “missing” including a sequence with homology to TNF-α. The published chTNF-α sequence (33) shows almost complete identity across most of the length with the sequence obtained in this study. However, the Bornelov et al. sequence is missing 216 nucleotides at the 5′ end and contains an insertion of 55 consecutive G nucleotides near the 3′ end (Figure S4 in Supplementary Material). These are, in our opinion, sequence assembly artifacts caused by the extremely high GC content. While only a small part of the sequence from the Bornelov et al. study was experimentally verified, we have confirmed the complete chTNF-α sequence by RT-PCR and sequencing. The chicken cytokine has an extended intracellular domain with unknown function at this point, as well as a conserved transmembrane sequence and displays the characteristic TNF superfamily motif in its extracellular part. The extracellular portion is 45% similar at the amino acid level to human TNF-α which is much higher than observed with other chicken cytokines, e.g., the interferons with only 20–25% similarity (12). The sequence of the intracellular domain is very distant to corresponding sequences of non-avian vertebrates and is quite variable even among avian species. We identified full or partial sequences with high homology to chTNF-α in 12 avian species including *Palaeognathae* and *Neognathae* supporting the concept that this cytokine is phylogenetically conserved across avian species. In mammals, the extracellular portion of TNF-α is cleaved by metalloproteinases such as TNF-α converting enzyme between the alanine^76^ and valine^77^ residues to release the soluble and biologically active form (45). This precise cleavage site is not present in the chicken sequence, while the relatively high overall sequence similarity points at an identical biology. To address this assumption, we cloned and expressed the extracellular part of chTNF-α and confirmed its biological activity using the NFκB-reporter assay (46) indicating that an alternative cleavage site should be present in the avian protein.

Phylogenetic analysis clustered the newly identified protein within the TNF-α family next to amphibian TNF-α and close to the human gene but more distant to the mammalian lymphotoxin genes and the fish TNF-α homologs. Earlier studies reported that TNF-α is missing in the chicken MHC locus (14) pointing at a translocation event of either the TNF-α gene or the entire syntenic locus. Screening of avian genome databases identified a contig in the recently published crow genome harboring the avian TNF-α sequence next to nine thus far mostly unknown genes, whose orthologs are ascribed to the mammalian MHC locus. Importantly, this contig did not contain LTα and LTβ orthologs as it would be expected based on synteny with mammalian genomes (47). This observation confirms the translocation of the TNF-α locus from the MHC region to a thus far unknown genomic location in birds and provides further evidence to the hypothesis that critical immune genes may be present in birds but have not been identified as a consequence of translocation events and the high GC contents. In this context, it should be noted that the lymphotoxins might be an exception and may indeed be absent in species without lymph nodes as suggested by others (12).

Previous work indicated the presence of orthologs of both receptors of chTNF-α (29, 34). We followed up on this work and obtained full sequences for both genes. Both receptors very closely cluster with their reptile orthologs and show close relation to the human and mouse receptors. Collectively, these studies show that the TNF-α/TNF-α receptor genes are encoded in the chicken and many avian genomes and might thus display similar biological functions.

In mammals, macrophages stimulated by TLR ligands are known to be the primary source for TNF-α (4). Likewise, chicken monocyte derived macrophages rapidly respond to LPS treatment with the induction of the TNF-α gene. Transcripts reached peak abundance within 2 h after stimulation and were at background levels at 24 h after treatment. This kinetic feature is similar to that reported in mice (48). The observed response pattern was independent of the macrophage source since splenic and BM-derived macrophages showed nearly identical kinetics. In mammals, T-lymphocytes are known as an additional source of TNF-α (49). Therefore, we isolated splenic lymphocytes, activated the cells through TCR α/β crosslinking and observed strong chTNF-α expression. This response was restricted to the CD4^+^ T-helper cell population which is in full agreement with results obtained in mouse and man (50). Next, we treated birds with LPS to confirm our *in vitro* observations. TNF-α mRNA was significantly induced in spleen but not in liver 3 h after treatment, while IL-6 was induced in both tissues and too much higher levels at this single time point. The lack of chTNF-α expression in the liver may be explained by its state of active tolerance to LPS which is constantly delivered from the gut *via* the portal vein as reviewed in Ref. (51). However, the comparatively weak induction of chTNF-α in splenic tissue was surprising taken into account that the murine spleen responds to LPS within 2–6 h with strong TNF-α induction (48). However, these observations are in line with earlier work by several groups who unsuccessfully attempted to induce high levels of chTNF-α like activity in chicken macrophages sufficient for purification to homogeneity and further molecular characterization (17, 52). Likewise, oral treatment of chickens with the TLR-7 ligand S-28463 induced a strong cytokine response in spleens with interferon, chemokine, and IL-6 mRNA expression, while expression of a TNF-α like message was not observed (53). The failure to identify a TNF-α ortholog in chickens prior to the availability of large genomic sequence databases may partially be due to comparatively low expression levels or inadequate stimuli used in earlier studies. Using the information provided here, more detailed analysis of the chTNF-α biology will now be possible including extended *in vitro* and *in vivo* induction studies and more detailed analysis of the induction kinetics.

Finally, we generated recombinant chTNF-α in eukaryotic and prokaryotic expression systems to demonstrate its biological activity. Upon binding to TNFR1, the cytokine induces a complex signaling cascade which ultimately leads to activation of the classical NFκB pathway (26) and NFκB induction (54). In a previous study, we established a reporter assay based on the quail fibroblast cell line CEC-32 by stably transfecting the cells with an NFκB-luciferase reporter. This cell line rapidly responds to rec. chIL-1 (46) which led us to assume that it may also be responsive to chTNF-α. As expected, full-length chTNF-α expressed in HEK 293 cells induced the reporter to the same level as rec. chIL-1. This effect was cytokine specific since (1) cell culture supernatants from mock transfected cells did not induce the reporter, (2) heat treatment abrogated the activity, and (3) LPS, as a potential contaminant to the culture supernatant, did not lead to a response over a concentration range from 0.1 ng/ml to 10 µg/ml. The reporter cell line used in this study is a quail cell line (55) which may not express TLR-4 in contrast to chicken primary macrophages, macrophage cell lines, and heterophils and may therefore be unresponsive to LPS (56). An identical response pattern was observed with *E. coli* expressed full-length chTNF-α and the extracellular domain of the cytokine in a series of independent experiments.

Based on the genomic and functional data presented in this study, we provide strong evidence for the existence of a true ortholog of TNF-α in birds. This work lays the foundation to gain new insights into the response of avian hosts to infection and inflammatory stimuli. Thus far, research on the inflammatory response in birds has mainly focused on the analysis of IL-1 and IL-6 (38, 53, 57, 58). Both cytokines are known to be induced by TNF-α (59, 60) in myeloid cells and, on the other hand, are capable of inducing this cytokine (61). The regulation of this intricate network could not be investigated in avian species to date but may help to understand unique responses of birds to pathogens. The availability of biologically active rec. chTNF-α provides a basis for functional studies *in vitro* and *in vivo*. Technologies such as *in vivo* overexpression or functional inhibition by retroviral vector-mediated gene transfer have been used successfully in previous research into the avian TNF family (62) and the type I IFN system (63, 64) and can be explored to further understand the chTNF-α/TNFR system. In addition, this work opens new avenues to investigate immunomodulatory properties of avian pathogens targeting the TNF family as shown in particular for herpes and pox viruses in mammals (65) and as previously studied for IFN modulatory factors in the chicken system (66, 67).

## Materials and Methods

### Animals

All chickens were hatched and housed at the Institute of Animal Physiology (University of Munich, Germany). Commercial diet and water were provided *ad libitum*. For LPS treatment, Lohmann Selected Leghorn (LSL) chickens obtained from LSL Rhein-Main (Berglern, Germany) were used at an age of 6 weeks. Six birds were i.v. treated with 10 µg LPS in PBS/kg BW. Control animals received PBS only. Three hours after injection animals were euthanized, liver and spleen tissues were obtained and stored in RNAlater (Sigma Aldrich, USA). Animal experiments were approved by the Government of Upper Bavaria, License number 55.2-1-54-2531-121-09.

Blood was collected from the jugular vein of 3–4 months old white leghorn chickens (line M11) obtained from the Institute of Farm Animal Genetics (Federal Research Institute for Animal Health, Neustadt, Germany), and experiments were approved by the Government of Upper Bavaria, License number 55.2-1-542532.0-60-2015.

### Cell Culture

HEK293T and HEK293 cells were cultured in RPMI 1640 (Biochrom, Germany) supplemented with 10% FBS (Biochrom, Germany) (standard medium) at 37°C and 5% CO_2_. Cells were transfected using ViaFect transfection reagent (Promega, Germany) according to the manufacture protocol. Supernatant was collected 48 h post-transfection and concentrated using an Amicon Ultra-15 Centrifugal Filter Units according to the manufacturer (Merck, UK).

CEC–NFκB–LUC cells (46) were cultured in Iscove’s basal Medium (Biochrom, Germany) supplemented with 8% FBS (Biochrom, Germany), 2% chicken serum (Thermo Scientific, Germany), 1% penicillin/streptomycin at 40°C, and 5% CO_2_.

### Isolation and Cultivation of Primary Chicken Macrophages

60 ml blood was collected into syringes containing 200 µl Li-Heparin (500 IU/ml in RPMI 1640, Biochrom, Germany). Peripheral blood mononuclear cells were separated by density gradient centrifugation over Biocoll (1,077, Biochrom, Germany) at 200 × *g* for 20 min. Cells were washed twice with PBS and 1 × 10^8^ cells in 10 ml standard medium were plated in cell culture treated Petri dishes (ϕ 9 cm) and incubated at 40°C and 5% CO_2_. After 72 h, plates were washed three times with PBS to remove non-adherent cells. Spleen macrophages were isolated according to the procedure described (68). BM macrophages were generated as described by Garceau et al. (36) using rec. chicken CSF-1 to differentiate BM precursors into macrophages. Macrophages adherent to cell culture Petri dishes were stimulated with LPS as indicated for the indicated periods. Purity of macrophage preparations was assessed by staining with monoclonal antibody KUL01-PE (SouthernBiotech, Birmingham, AL, USA) at a 1:100 dilution and live-dead cell discrimination was obtained with fixable viability dye eFluor^®^ 780 (Thermo Fisher Scientific) followed by analysis using a BD FACSCanto II (Becton Dickinson, Heidelberg, Germany) and FACS Diva software.

### Isolation of Lymphocytes and Cell Sorting

Leukocytes were isolated from spleen tissue as described (37) and either activated without further separation or as purified CD4^+^ or CD8^+^ cell preparations. Purification was achieved by cell sorting with a BD FACSAriaIIIu (Becton Dickinson, Heidelberg, Germany). Monoclonal antibodies reacting with chicken CD8α (clone 3-298) (69) or chicken CD4 (CT4, SouthernBiotech, Birmingham, AL, USA) were used as FITC or RPE conjugates, respectively, to directly label T-cell subpopulations. Lymphocyte activation was achieved by TCR crosslinking as described (37).

### mRNA Isolation, cDNA Synthesis, and PCR Amplification

Total RNA was isolated using TRI reagent (Sigma-Aldrich, St. Louis, MO, USA) from lung tissue of Brown Leghorn chicken. Reverse transcription was performed using the SMART RACE (Clontech, Palo Alto, CA, USA) procedure, 1 µg RNA, and Moloney murine leukemia virus reverse transcriptase (NEB, Ipswich, MA, USA). To amplify the highly GC-rich TNF-α gene, previously reported conditions were used (31): a 1:200 mixture of Deep Vent and Taq polymerases (both from NEB) and long (5–10 min) extension times. The TNF-α cDNA was divided into five overlapping short amplicons, which cover the entire coding sequence. The primer pairs used were 5′-CCATATGACCACGCTCTTTCCGT and 5′-AGCAGCAGCAGCAGCAGAGC, 5′-GTGGGCGGTGCGGCCATA and 5′-ACGTCGTTCTGAGCGGAGCTGT, 5′-GGGGAGAGGACCCCACAGCTC and 5′-CCTTCTCAGCACCACGCCGTTA, 5′-GTCCTCTCAGCCCGGCTCGTT and 5′-GGTCAGGAGGGGGACGTCTTTG, 5′-CCGGGACGGCCTTTACTTCGTA and 5′-CTAATTTAATCCACTCCCACCACCC. The PCR products were directly sequenced following isolation from agarose gel electrophoresis.

For gene expression studies, total RNA was isolated from LPS treated and non-treated macrophages using Trifast (peqGOLD TriFast, Peqlab, Germany) according to the manufacturer’s protocol. Tissues were homogenized using a Precellys^®^ tissue homogenizer (Peqlab, Germany) prior to RNA isolation. 1 µg total RNA was treated with DNase I, RNase-free-kit (Thermo Scientific, Germany) to remove genomic DNA. cDNA was synthesized from 400 ng digested RNA using GoScript™ Reverse Transcription System kit as recommended by the manufacturer (Promega, Germany).

### Cloning chTNF-α Expression Construct

The chTNF-α nucleotide sequence was codon optimized for expression in human cell lines to avoid GC-rich regions using the online tool provided by GeneArt (Thermo Scientific, Germany). The codon-optimized sequence was used to synthesize chTNF-α and to clone it into the expression vector pcDNA3.1. Gene synthesis and cloning were done using GeneArt. The plasmid sequence was confirmed by sequencing.

The chTNF-α sequence from the expression vector pcDNA3.1 was cloned into pET-45b(+) (Novagen^®^, Merck, UK) using Q5^®^ High-Fidelity DNA Polymerase (NEB, Germany) and the following overhang primers: full length forward (5′-CACCACCACCATCACGTGGGTACCGGTACCACCCTGTTCCCTTGC), full length reverse (5′-AGCGGTTTCTTTACCAGACTCGAGTCAATCCACTCCCACGAC), extracellular domain forward (5′-TCACCACCACCATCACGTGGGTACCGGTCCTTGGGGCGAGGATCCT), and extracellular domain reverse (5′-CAGCGGTTTCTTTACCAGACTCGAGTCAATCCACTCCCACGACTC).

The DNA was purified using the Wizard^®^ SV Gel and PCR Clean-Up System (Promega, Germany) according to the manufacturer instructions.

Purified DNA fragments and pet45b vector cut with AgeI and XhoI (Thermo Scientific, Germany) and assembled using NEBuilder^®^ HiFi DNA Assembly Master Mix (NEB) according to the manufactures protocol. The final constructs were then transformed into *E. coli* NEB5alpha cells using a 42°C heat-shock for 30 s. Properly transformed cells were selected over ampicillin resistance. The plasmids were isolated using the PureYield™ Plasmid Miniprep System Technical Bulletin (Promega, Germany) and were verified by sequencing.

### Expression of Recombinant chTNF-α

To express recombinant chTNF-α, we transformed the pet45b vector including either the full length or the extracellular chTNF-α into *E. coli* BL21 (DE3) competent cells using the manufacturers protocol (Biolabs, USA). Shortly, BL21 cells were thawed on ice, the plasmid DNA was added and transformed by heat-shock for 10 s at 42°C. After successful selection, the protein was produced from 2 l bacteria suspension after induction of chTNF-α expression with 50 µM IPTG. Cells were centrifuged and the resulting cell pellet was suspended in ultrasonic buffer (300 mM NaCl, 50 mM Na_2_HPO_4_, 1 mM DTT, pH 7.8). Cells were lysed by ultrasound seven times for 30 s while kept on ice. Cells were again centrifuged and the pellet was suspended in lysis buffer (6 M guanidine-HCl, 100 mM NaH_2_PO_4_, 10 mM Tris–HCl, 1 mM DTT, pH 8.0), centrifuged and the supernatant collected. Ni-NTA Agarose (Qiagen, Germany) was washed once with water and once with the lysis buffer and mixed with the supernatant for overnight rotation at 4°C. The mixture was added to a column and washed with lysis buffer and subsequently with washing buffer 1 (8 M urea, 100 mM Na_2_HPO_4_, 10 mM Tris–HCl, 1 mM DTT, pH 8.0) and washing buffer 2 (2 M urea, 1 M NaCl, 100 mM Na_2_HPO_4_, 10 mM Tris–HCl, 1 mM DTT, pH 8.0). To eluate the protein, two elution buffers with different pH were used. First buffer contained 50 mM Na-acetate, 1 M NaCl, 2 M urea, 10 mM Tris–HCl, 1 mM DTT, pH 6.5, added to the column and let flow through until 3 ml were left. Then the second elution buffer (50 mM Na-acetate, 1 M NaCl, 2 M urea, 1 mM DTT, pH 3.6) was added and the protein was eluted with pressure and collected in several fractions. All fractions were then tested for the specific biological activity using the luciferase bioassay.

### Quantitative RT-PCR

Quantitative RT-PCR was performed using either SYBRGreen (GoTaq^®^ qPCR Master MixPromega, Germany) or TaqMan (Biolabs, USA) chemistry. Target gene expression was normalized to the expression of 18S rRNA as previously described (70). For qPCR analysis, the following primers were used: 18S rRNA: 5′-CATGTCTAAGTACACACGGGCGGTA and 5′-GGCGCTGCTGGCATGTATTA; chTNFα: 5′-CGCTCAGAACGACGTCAA and 5′-GTCGTCCACACCAACGAG; chIL-6: 5′-GCTTCGACGAGGAGAAATGC and 5′-GCCAGGTGCTTTGTGCTGTA; chIFN-γ: 5′-TGGCGTGAAGAAGGTGAAAGA and 5′-TCCGCAGCTGGAAAAAGTG. ChTNF-α primers were designed using Primer 3 Software (71) and all primers were purchased from Eurofins (Germany). The reactions were performed using a 7300 Real Time PCR System (Applied Biosystems, Thermo Scientific).

### Bioassay for chTNF-α and LPS Quantification

CEC–NFκB–LUC cells were seeded into 96-well plates at a density of 1 × 10^5^ cells per well and incubated for 12–16 h. After washing the cells with PBS, concentrated cell culture supernatant containing chTNF-α or Ni-Agarose purified TNF-α was added to each well and cultures were incubated for another 6 h. The following steps including cell lysis and analysis were performed according to the manufacturer’s protocol (Luciferase Assay system, Promega, Germany). Samples were measured on a Glomax 96 microplate luminometer (Promega, Germany).

Lipopolysaccharide concentrations in chTNF-α preparations were quantified by LAL assay as described by the manufacturer (Thermo Scientific, Germany).

### Mass Spectrometry Analysis

Stably transfected HEK293T cells, cultivated as mentioned above, were washed six times with serum-free RPMI medium. B&S serum-free medium (Bio&Sell, Germany) was applied for 6 h and supernatant was collected and concentrated using Amicon Ultra-15 Centrifugal Filter Units. Protein aliquots (1 mg) were solubilized using sodium deoxycholate [1% (w/v) final conc.], reduced with TCEP [tris(2-carboxyethyl)phosphine], alkylated with MMTS (S-methyl methanethiosulfonate), digested sequentially with trypsin and extracted with ethylacetate saturated with water as described (72). Samples were desalted on OPTI-TRAP Macro (Optimize Technologies, Oregon City, OR, USA), dried in Speedvac and dissolved in 20 mM ammonium formate + 2% acetonitrile. About 1 mg of peptide digests were separated on C18 column (Kinetex 1.7 µm, EVO C18), with linear gradient from 0% A (of 20 mM ammonium formate, 2% acetonitrile pH = 10) to 50% B (of 20 mM ammonium formate, 80% acetonitrile pH = 10) in 32 min, flow 300 µl/min. 32 fractions were collected and pooled into 8 fractions (73); the resulting fractions were dried and resuspended in 20 µl of 1% trifluoroacetic acid. About 2 µg of peptide from each fraction were separated on 50-cm C18 column using 2.5 h elution gradient and analyzed in a DDA mode on Orbitrap Fusion Tribrid (Thermo Scientific) mass spectrometer. Resulting raw files were processed in MaxQuant (v. 1.5.8.3) (74). Searches were performed against latest version of human Uniprot reference database, sequence of protein of interest, and common contaminant database. Downstream analysis and visualization was performed in Perseus (v. 1.5.5.3) (75).

### Western Blot Analysis

Purified chTNF-α, diluted 1:6 into Lämmli buffer containing additional 0.5 µl 1 M DTT (Thermo Scientific, USA), was separated on a 10% SDS-PAGE gel and transferred to a nitrocellulose membrane (*Amersham Protran*^®^ 0.2 µm, GE Healthcare Europe GmbH, Germany) using the Mini Trans-Blot Electrophoretic Transfer Cell (BioRad, Germany) at 100 V for 1 h. The membrane was blocked with 4% skim milk (Applichem, Germany) for 1 h, washed three times with PBS-T and incubated with an anti-His-antibody (0.2 mg/ml, Dianova, Germany) 1:1,000 diluted in PBS-T for 1 h. After three washings, Goat-Anti-Mouse IgG (H + L)-HRPO (©Jackson ImmunoResearch, USA) at a 1:10,000 dilution was added for 1 h and membranes were subsequently washed six times. Membranes were developed with Luminol reagent (Sigma Aldrich, USA) for 1 min and images were taken using the Image Quant Capture 300 device (GE Healthcare Europe GmbH, Germany). An irrelevant HIS-tagged protein was used as a control.

### Computational Analysis of Avian Sequences From SRA

To assemble the chTNF-α sequence, several large datasets from the NCBI SRA were used (mainly Illumina RNA-seq studies ERP003988, SRP026393, SRP033603, and SRP014719). Sequences of non-avian vertebrate TNF-α genes were used as probes in BLAST searches of the SRA datasets. The sequences obtained were downloaded and assembled manually either with CLC genomics workbench 8.0.1 (www.clcbio.com) or with Lasergene 10.0.0 (DNASTAR, Madison, WI, USA). The resulting short contigs were used as probes in subsequent rounds of BLAST searches against SRA datasets, until the full open reading frame was completed. To obtain TNF-α from other avian species, the chicken gene was used as a probe to interrogate by BLAST the SRA RNA-seq or genomic datasets from the particular species. In some cases, the coverage of the TNF-α gene was not sufficient to assemble the complete coding sequence.

### Sequence Alignment and Phylogenetic Inference

Amino acid sequences were aligned using the MAFFT software v7.271 with L-INS-i algorithm (76). Prior to the phylogenetic inference, columns with more than 80% of gaps were discarded. Maximum likelihood phylogeny was generated using MEGA 6 software (77). WAG + F model with gamma distribution (six categories) of rates among sites was used as a best-fitting substitution model (according to the Akaike Information Criterion calculated in Smart Model Selection module of PhyML software). The SPR operations in an optimized BioNJ starting tree were used for searching of the final tree. Bootstrap support for each node was evaluated with 1,000 replicates.

### Statistical Analysis

Statistics were performed using SPSS 24 (IBM, USA) calculating either the Student’s *t*-test or Mann–Whitney *U*-test.

## Ethics Statement

Animal experiments were approved by the Government of Upper Bavaria, License number 55.2-1-54-2531-121-09. Blood sampling was approved by the Government of Upper Bavaria, License number and 55.2-1-542532.0-60-2015.

## Author Contributions

FR planned and performed experiments, analyzed data, and wrote the paper. BS planned experiments and wrote the paper. TH performed bioinformatic data analysis and phylogenetic analysis. HF performed experimental work on gene expression and sequencing. JP performed preliminary *in vivo* experimental work. SH isolated lymphocytes and performed cell sorting experiments. JH analyzed results, contributed to writing the final version of the manuscript. DE performed bioinformatic data analysis and phylogenetic analysis, evaluated the experiments, participated in drafting the manuscript. BK planned the project and experiments, analyzed data, and wrote the paper. All authors contributed to manuscript revision, read and approved the submitted version.

## Conflict of Interest Statement

The authors declare that the research was conducted in the absence of any commercial or financial relationships that could be construed as a potential conflict of interest.

## Supplementary Material

Figure S1 Alignment of TNF-α amino acid sequences from various avian species. In some avian species, only partial TNF-α sequence could be obtained from the available National Center for Biotechnology Information Sequence Read Archive data. In such cases, the 5′ and/or 3′ regions are filled with blue “X” letters in the alignment to indicate the missing parts. In addition to the avian species mentioned in Figure 2, the following species are included: rock pigeon (*Columba livia*), golden eagle (*Aquila chrysaetos*), mallard (*Anas platyrhynchos*), swan goose (*Anser cygnoides*), African ostrich (*Struthio camelus*), brown kiwi (*Apteryx mantelli*), and white-throated tinamou (*Tinamus guttatus*). The protein domain positions and the TNF superfamily motif are shown above the alignment.

Figure S2 Comparison of GC content and the presence of GC-rich stretches in avian and non-avian TNF-α genes. The GC content is plotted against the average length of GC-rich stretch, which is defined as an uninterrupted sequence of at least three consecutive G or C nucleotides. For comparison with the bulk of the chicken genes (all approximately six thousand chicken RefSeq coding sequences longer than 299 nucleotides), a histogram of the distribution of GC-rich stretches in the chicken RefSeq gene category is shown as a grey line. The TNF-α genes from the following species were included: avian species shown in the tree in Figure 2; non-avian species shown in the alignment in Figure 1; channel catfish and common carp.

Figure S3 Mass spectrometry and Western blot analysis of the recombinant chicken TNF-α (chTNF-α). Spectra of three unique peptides of the recombinant chTNF-α protein produced in HEK293 cell supernatant. The peptide sequence is shown below each diagram **(A)**. Western blot analysis to detect the extracellular chTNF-α (line 2) at the assumed level of 21 kDa. The Protein weight was calculated using the Editseq software (DNASTAR^®^, USA). Line 1 shows the HIS-tagged protein which was used as a positive control for the Tag **(B)**.

Figure S4 Pairwise alignment of a previously published chicken TNF-α (chTNF-α) nucleotide sequence with the sequence reported in this study. The full coding sequences of chTNF-α from this study and from the work of Bornelov et al. (33) were used in the alignment. The predicted transmembrane domain is shown in a gray box. The extent of sequence confirmed by RT-PCR and sequencing in the Bornelov et al. study is depicted by blue line.
