# Supplementary figures and images for: Characterization of Chicken Tumor Necrosis Factor-α, a Long Missed Cytokine in Birds
Source: Front Immunol. 2018 Apr 17;9:605. doi: 10.3389/fimmu.2018.00605 (PMC5913325; doi:10.3389/fimmu.2018.00605)

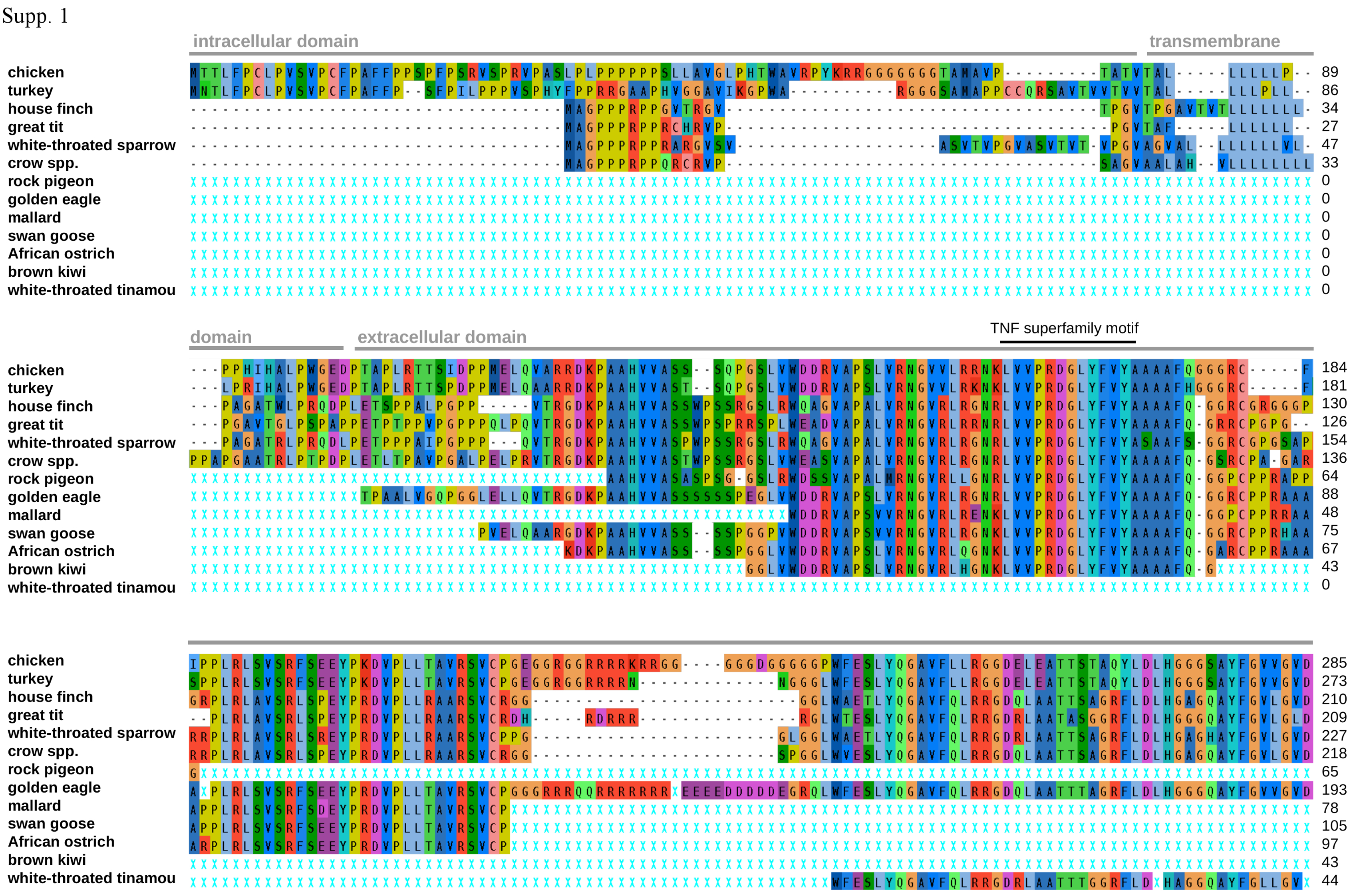

Supplement: Figure S1 — Alignment of TNF-α amino acid sequences from various avian species. In some avian species, only partial TNF-α sequence could be obtained from the available National Center for Biotechnology Information Sequence Read Archive data. In such cases, the 5′ and/or 3′ regions are filled with blue “X” letters in the alignment to indicate the missing parts. In addition to the avian species mentioned in Figure 2, the following species are included: rock pigeon (Columba livia), golden eagle (Aquila chrysaetos), mallard (Anas platyrhynchos), swan goose (Anser cygnoides), African ostrich (Struthio camelus), brown kiwi (Apteryx mantelli), and white-throated tinamou (Tinamus guttatus). The protein domain positions and the TNF superfamily motif are shown above the alignment. [file image_1.jpeg]

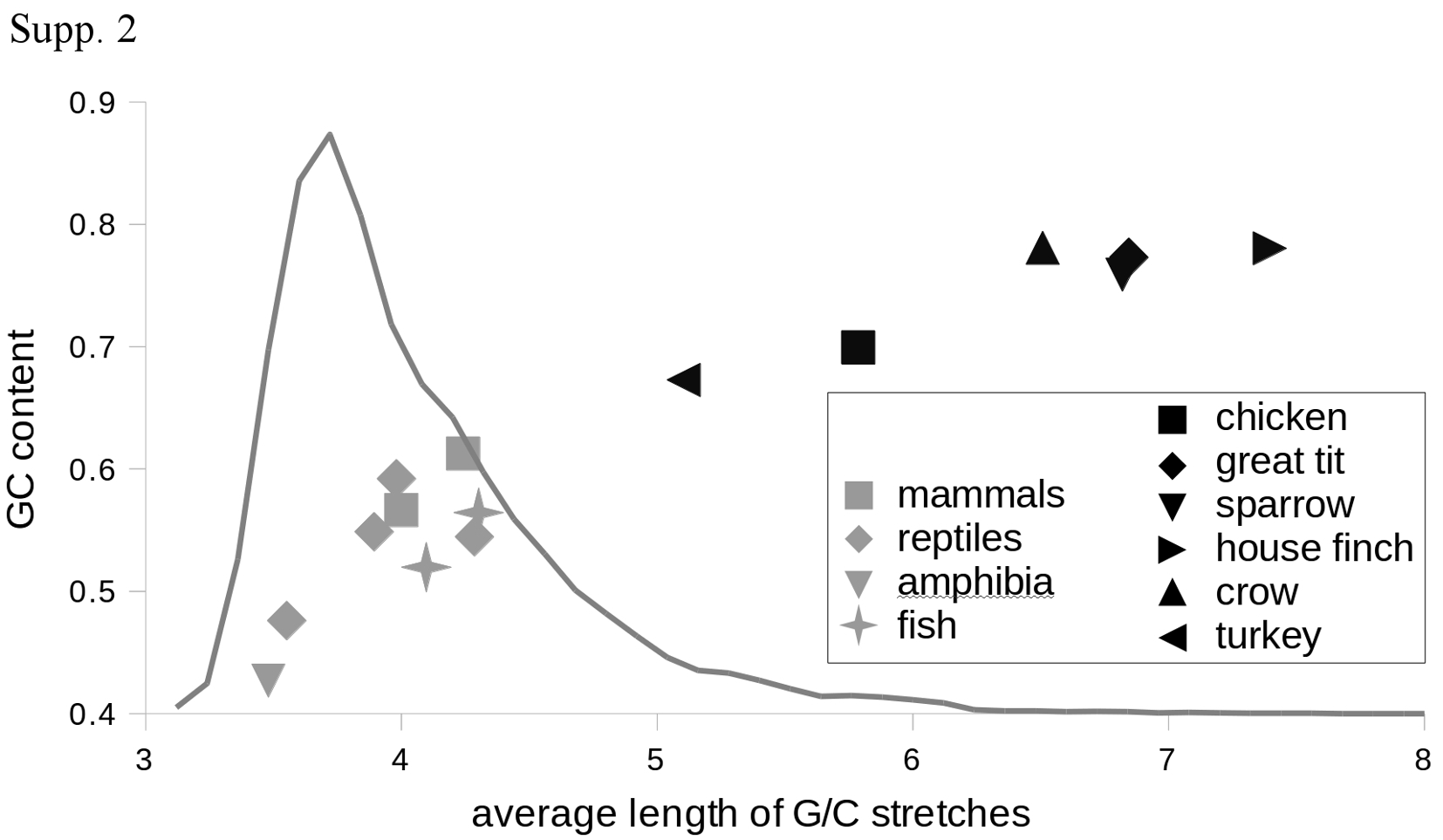

Supplement: Figure S2 — Comparison of GC content and the presence of GC-rich stretches in avian and non-avian TNF-α genes. The GC content is plotted against the average length of GC-rich stretch, which is defined as an uninterrupted sequence of at least three consecutive G or C nucleotides. For comparison with the bulk of the chicken genes (all approximately six thousand chicken RefSeq coding sequences longer than 299 nucleotides), a histogram of the distribution of GC-rich stretches in the chicken RefSeq gene category is shown as a grey line. The TNF-α genes from the following species were included: avian species shown in the tree in Figure 2; non-avian species shown in the alignment in Figure 1; channel catfish and common carp. [file image_2.jpeg]

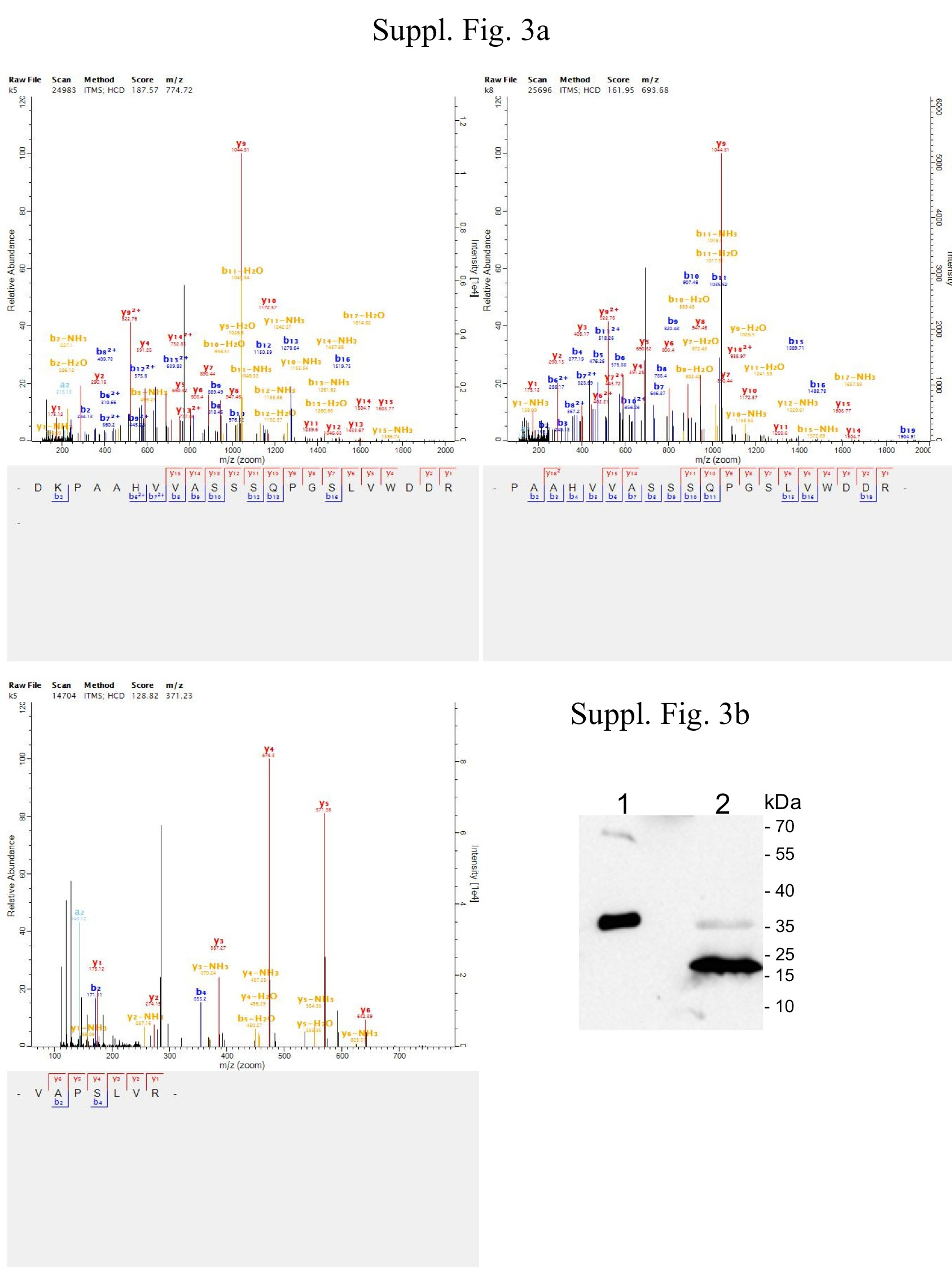

Supplement: Figure S3 — Mass spectrometry and Western blot analysis of the recombinant chicken TNF-α (chTNF-α). Spectra of three unique peptides of the recombinant chTNF-α protein produced in HEK293 cell supernatant. The peptide sequence is shown below each diagram (A). Western blot analysis to detect the extracellular chTNF-α (line 2) at the assumed level of 21 kDa. The Protein weight was calculated using the Editseq software (DNASTAR®, USA). Line 1 shows the HIS-tagged protein which was used as a positive control for the Tag (B). [file image_3.jpeg]

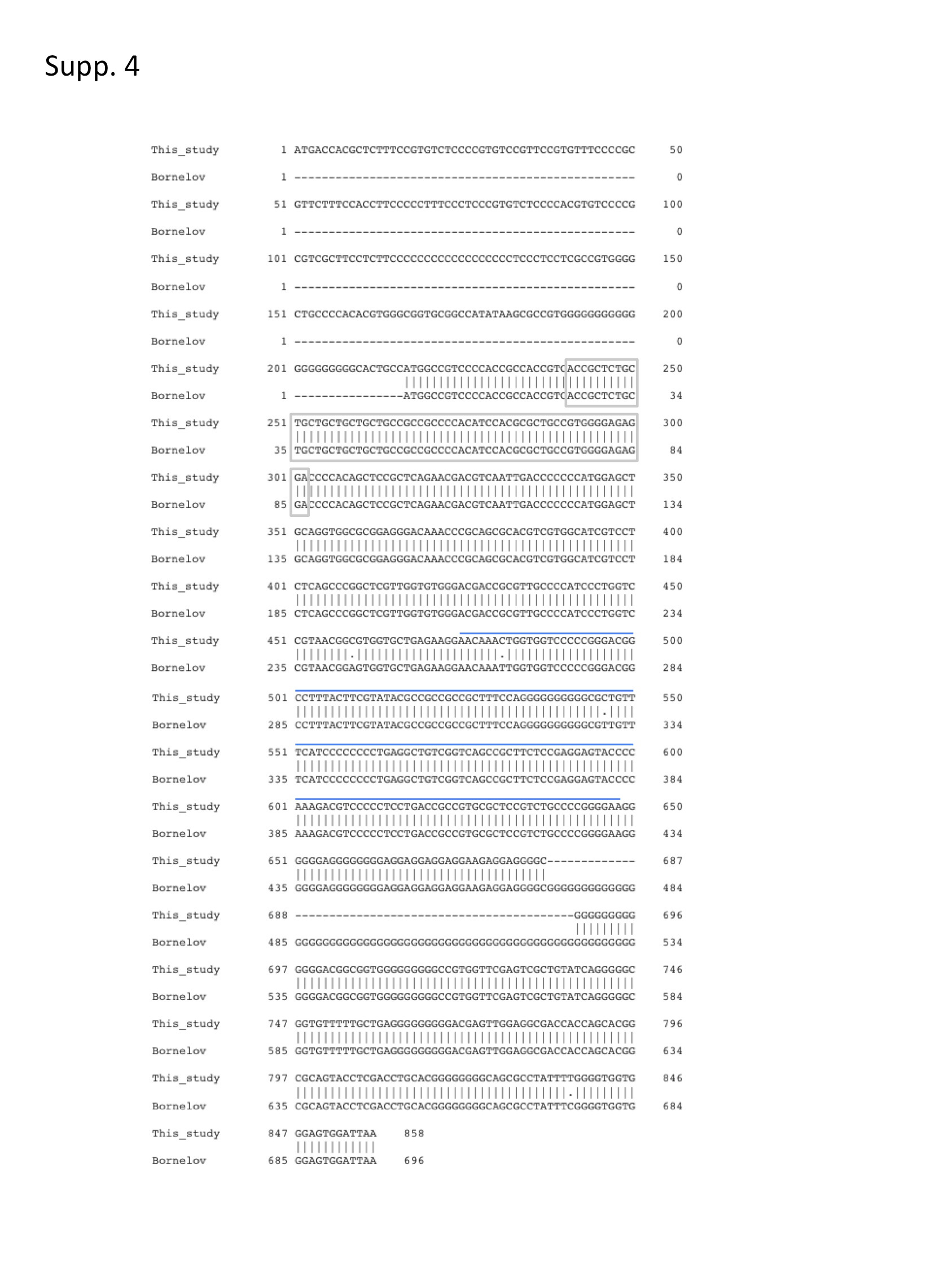

Supplement: Figure S4 — Pairwise alignment of a previously published chicken TNF-α (chTNF-α) nucleotide sequence with the sequence reported in this study. The full coding sequences of chTNF-α from this study and from the work of Bornelov et al. (33) were used in the alignment. The predicted transmembrane domain is shown in a gray box. The extent of sequence confirmed by RT-PCR and sequencing in the Bornelov et al. study is depicted by blue line. [file image_4.jpeg]
